# An automated detection of epileptic seizures EEG using CNN classifier based on feature fusion with high accuracy

**DOI:** 10.1186/s12911-023-02180-w

**Published:** 2023-05-22

**Authors:** Wenna Chen, Yixing Wang, Yuhao Ren, Hongwei Jiang, Ganqin Du, Jincan Zhang, Jinghua Li

**Affiliations:** 1 The First Affiliated Hospital, and College of Clinical Medicine of Henan University of Science and Technology, Luoyang, China; 2 College of Information Engineering, Henan University of Science and Technology, Luoyang, China

**Keywords:** Epileptic EEG signal classification, Random forest, Convolutional neural networks, Epilepsy detection, Feature selection

## Abstract

**Background:**

Epilepsy is a neurological disorder that is usually detected by electroencephalogram (EEG) signals. Since manual examination of epilepsy seizures is a laborious and time-consuming process, lots of automatic epilepsy detection algorithms have been proposed. However, most of the available classification algorithms for epilepsy EEG signals adopted a single feature extraction, in turn to result in low classification accuracy. Although a small account of studies have carried out feature fusion, the computational efficiency is reduced due to too many features, because there are also some poor features that interfere with the classification results.

**Methods:**

In order to solve the above problems, an automatic recognition method of epilepsy EEG signals based on feature fusion and selection is proposed in this paper. Firstly, the Approximate Entropy (ApEn), Fuzzy Entropy (FuzzyEn), Sample Entropy (SampEn), and Standard Deviation (STD) mixed features of the subband obtained by the Discrete Wavelet Transform (DWT) decomposition of EEG signals are extracted. Secondly, the random forest algorithm is used for feature selection. Finally, the Convolutional Neural Network (CNN) is used to classify epilepsy EEG signals.

**Results:**

The empirical evaluation of the presented algorithm is performed on the benchmark Bonn EEG datasets and New Delhi datasets. In the interictal and ictal classification tasks of Bonn datasets, the proposed model achieves an accuracy of 99.9%, a sensitivity of 100%, a precision of 99.81%, and a specificity of 99.8%. For the interictal-ictal case of New Delhi datasets, the proposed model achieves a classification accuracy of 100%, a sensitivity of 100%, a specificity of 100%, and a precision of 100%.

**Conclusion:**

The proposed model can effectively realize the high-precision automatic detection and classification of epilepsy EEG signals. This model can provide high-precision automatic detection capability for clinical epilepsy EEG detection. We hope to provide positive implications for the prediction of seizure EEG.

## Introduction

Epilepsy is the second most common neurological disorder after stroke, according to a report from World Health Organization [1, 2]. People with epilepsy account for about 1% of the world population. Due to the uncertainty of ictal, epilepsy patients need to take long-term medication, which brings great harm to their bodies and mind. Therefore, the analysis and mining of epilepsy features are helpful to achieve early warning of epileptic seizures, which can not only ensure the personal safety of patients, but also remind patients to choose emergency antiepileptic drugs. The development of electroencephalogram (EEG) has prompted the emergence of a low-cost, high-efficiency EEG recognition technology for epilepsy [3]. The EEG features of epileptic patients and normal people are quite different. EEG activity in patients with epilepsy is usually divided into interictal and ictal phases, and there are significant differences in EEG features between interictal and ictal. The way that neurosurgeons read EEG signals to determine if people have epilepsy is a general approach in the medical community. However, the observation and detection of EEG signals is a time-consuming and laborious task [4]. Not only does it require many manpower and material resources, but also has a high risk of misdiagnosis. Therefore, the automatic detection and classification model of EEG signals is becoming more and more urgent.

In recent years, in order to realize the automatic diagnosis of epilepsy EEG signals, various automatic detection and classification models have been proposed. In order to extract the features of EEG signals effectively, the decomposition of the signal is required to be performed first. Since the wavelet transform can handle non-smooth and complex signals such as EEG signals while the traditional Fourier transform used for time–frequency domain analysis of signals can only handle smooth signals, a large number of studies have employed Discrete Wavelet Transform (DWT) to decompose EEG signals [5–7]. Furthermore, analyzing and extracting the effective signal features play an important role in classification, to realize the automatic detection of epilepsy. However, only a single feature was adopted for EEG classification in most of the available studies for epilepsy EEG detection. In general, the features which are used to detect epilepsy contain the following categories: Power Spectral Density Energy Diagram (PSDED) represented by energy analysis [5], nonlinear characteristics Approximate Entropy (ApEn), Distribution Entropy (DistEn), Shannon Entropy (ShanEn), Renyi Entropy (RenEn) and LempelZiv Complexity [8–15], and Common Spatial Pattern (CSP) algorithms for the spatiotemporal domain [16]. A single EEG feature can only describe part of the EEG features, resulting in poor classification accuracy. Yet, the combination of the above features can better reflect the features of EEG signals in epilepsy. For example, some studies combine various nonlinear features such as Hurst Exponent (HE), Kolmogorov Complexity (KC), ShanEn, and Sample Entropy (SampEn) [15, 17, 18], and a fusion of spatial and temporal features could also be performed [19]. However, if too many epileptic EEG features are extracted and fused, it may lead to lower computational efficiency and information redundancy, and there are also some bad features that interfere with the classification results. Therefore, a small number of studies have performed the selection of hybrid features, such as features selection by use of genetic algorithms based on the Viral Swarm Particle Optimization (VSPO) technique [20], but the classification accuracy obtained by this method is not high. In addition, according to the EEG characteristics of epilepsy, selecting an effective classification model is very critical for the automatic detection of epilepsy. With the development of artificial intelligence, machine learning models were widely used in automatic epilepsy detection, such as Artificial Neural Networks (ANN) [5], Random Forests (RF) [21], and Support Vector Machines (SVM). Although the traditional machine learning algorithms such as SVM are widely used, the method is more suitable for single channel and small sample datasets [13, 20, 22–24]. However, when larger data with multiple features for EEG signals is analyzed, deep learning algorithms such as Convolutional Neural Network (CNN) have obvious advantages compared to traditional machine learning algorithms [8, 19, 25–28].

To address the above multi-feature extraction and screening problems as well as to consider the performance of the used classifier, an automatic epileptic EEG signal recognition method based on feature fusion and selecting is proposed in this paper. Firstly, the EEG signal was decomposed by DWT, and the Joint Time–Frequency Analysis (JTFA) and nonlinear analysis were used to extract the EEG hybrid features of epilepsy. Secondly, the random forest algorithm was used to select some important features. Finally, CNN was used to classify the EEG signals. The structure of this article is as follows. The previous related works are investigated and summarized in Section II. Section III shows the dataset used in this experiment, in addition to describing the methods and algorithms used to establish the model in this paper. Section IV shows the experiment results and analysis. Finally, Section V concludes the paper by summarizing the contributions.

## Literature survey

Many automated epileptic EEG signal classification systems using a single feature have emerged in recent years. In EEG signals, features can be divided into time domain, frequency domain, time–frequency domain, and nonlinear features. Nonlinear features are often used in the classification of EEG signals [8, 10, 12, 13]. G. R. Kiranmayi and Udayashankara [8] proposed a method for nonlinear analysis of EEG based on ApEn feature, and the ApEn feature was extracted from the *δ*, *θ*, *α*, *β,* and *γ* subbands of healthy EEG, ictal and interictal EEG. Emran Ali et al. [10] analyzed and compared the effectiveness of DistEn, ShanEn, RenEn, and LempelZiv Complexity as classification features of seizures in EEG signals. Si Thu Aung et al. [12] proposed a modified Distribution Entropy (mDistEn) for epilepsy detection and obtained 92% classification accuracy by exploring the advantages of Fuzzy Entropy (FuzzyEn) and DistEn. Deepti Tripathi et al. [13] described the classification of EEG signals into healthy, interictal, and ictal using the EMD-based FuzzyEn method.

Shasha Zhang et al. [26] presented a lightweight solution. For the first stage, Pearson correlation coefficients are computed to obtain the correlation matrix. For the second stage, a simple CNN model was used to classify the correlation matrix to distinguish pre-episode states from inter-episode states with a prediction accuracy of 89.98%.

Aayesha et al. [29] proposed a fuzzy-based seizure detection model that incorporates a new feature extraction and selection method. For the binary classification problem of interictal and ictal periods, the classification accuracy rate of 96.67% was reached.

With the study of EEG characteristics, energy analysis of EEG signals and space–time analysis have emerged [9, 16, 30]. Yunyuan Gao et al. [9] proposed a deep learning-based method for the detection of epileptic EEG signals, where the epilepsy EEG signals were converted into Power Spectral Density Energy Daps (PSDED), which are then applied to Deep Convolutional Neural Networks (DCNNs) and transfer learning PSDED. N. Sriraam et al. [30] utilized Teager energy features to automatically detect seizures from multichannel EEG recordings and evaluated the performance of a multilayer perceptron neural network classifier using sensitivity, specificity, and false detection rate. Turky N. Alotaiby et al. [16] used the CSP algorithm to extract spatiotemporal domain features from EEG signals for the classification of EEG signals.

Rishabh Bajpai et al. [25] applied the spectrum to convert EEG signals into the image domain. The spectral images were then applied to CNN to learn robust features, which facilitate the automatic detection of pathological and normal EEG signals with experimental accuracy, sensitivity, and specificity of 96.65%, 90.48%, and 100%, respectively.

Zhao and Wang [31] proposed SeizureNet, a CNN-based model for robust seizure detection of EEG signals. Firstly, two convolutional neural networks were employed to extract time-invariant features from single-channel EEG signals. Secondly, the fully connected layer was used to learn the high-level features. Finally, these features were fed to the softmax layer for classification. They evaluated the model on a benchmark database provided by the University of Bonn, and a tenfold cross-validation method was used, obtaining up to 98.5% accuracy and 97.0% sensitivity for dichotomous mission between interictal and ictal period.

As seen from the above experiments, the classification accuracy obtained from a single feature is low. Therefore, some other studies performed feature fusion. Many researchers choose to fuse nonlinear features with other features [15, 17, 22].Mohd Syakir Fathillah et al. [15] combined multiple features such as HE, KC, ShanEn, and SampEn for EEG signals by studying multi-resolution analysis algorithms. Daniel Abásolo et al. [17] analyzed EEG recordings from patients with focal epilepsy using two nonlinear methods of ApEn and LempelZiv complexity. Yanan Lu et al. [22] combined three features to classify single-channel EEG signals for seizure detection, and the three features contain the Kraskov entropy feature based on the Hilbert-Huang Transform (HHT), the instantaneous area of the analytical eigenmode function of EEG signals, and the Kraskov entropy applied to the tunable Q wavelet transform, while the Least Squares Support Vector Machine (LS-SVM) classifier was used to classify the multivariate feature combination.

Sharma et al. [23] used the Empirical Modal Decomposition (EMD) method to decompose EEG signals and extracted the Intrinsic Mode Function (IMF). The entropy features of different IMFs for focal and nonfocal EEG signals were calculated, namely average Shannon Entropy (ShanEnAvg), average Renyi Entropy (RenEnAvg), average ApEn (ApEnAvg), average Sample Entropy (SampEnAvg) and average phase entropy (S1Avg and S2Avg). These entropies were used as input feature sets for LS-SVM classifiers to classify EEG signals into focal and nonfocal signals and the model achieved an average classification accuracy of 87%.

In addition, some researchers integrate temporal features with frequency-domain features or spatial features [19, 21, 24]. Hisham Daoud et al. [19] used DCNN and Bi-LSTM networks to learn important spatial and temporal features from raw data, respectively, and used a semi-supervised learning method based on DCAE with migration learning techniques for dichotomous classification of EEG states. Xiashuang Wang et al. [21] presented an automatic seizure detection model based on the method of multiple time–frequency analysis, which involves a new random forest model combined with grid search optimization. Abeg Kumar Jaiswal et al. [24] proposed an automatic detection method for EEG signal epilepsy based on subpattern Principal Component Analysis (SpPCA) and cross-subpattern correlation Principal Component Analysis (SubXPCA) combined with SVM.

Banupriya and Devi [20] used a genetic algorithm based on Virus Swarm Particle Optimization (VSPO) technique for feature selection and SVM technique for classification of EEG signals. The experimental results shown that the sensitivity was 98.03%, the specificity was 98.01%, and the accuracy was 98.90%.

Deivasigamani et al. [32] presented a computer-assisted method for automatic detection and classification of focal and nonfocal EEG signals. The Double-Tree Complex Wavelet Transform (DT-CWT) was used to decompose EEG signals and extract features from the decomposition coefficients. These features were trained and classified using the Adaptive Neural Fuzzy Inference System (ANFIS). Finally, the classification results with sensitivity of 98%, specificity of 100% and accuracy of 99% were obtained.

## Methods and materials

### Dataset

#### Bonn EEG dataset

The dataset used in this study is the epilepsy EEG dataset of the University of Bonn, Germany [33], which was collected from five healthy subjects and five epilepsy patients, and the dataset is a single-channel EEG signal dataset, containing five subsets (Set A ~ Set E). Each subset contains 100 data segments of the same type, and each data segment contains 4097 EEG time series. Each data segment has a time length of 23.6 s with a sampling frequency of 173.61 Hz, and the artifacts have been removed by manual filtering of 0.53 ~ 40 Hz. The electrode positions of Set A and Set B subsets were located on the scalp, which is the EEG data of 5 healthy subjects in the state of opened and closed eyes, respectively. The EEG data of Set C and Set D subsets were obtained from 5 epilepsy patients in the interictal period, while the electrode position of the Set C subset was located in the contralateral region of the lesion, and the electrode position of the Set D subset was located in the lesion area. The electrode position of the Set E subset was located in the lesion area, which is the EEG data of 5 epilepsy patients during the ictal period.

**Fig. 1:**
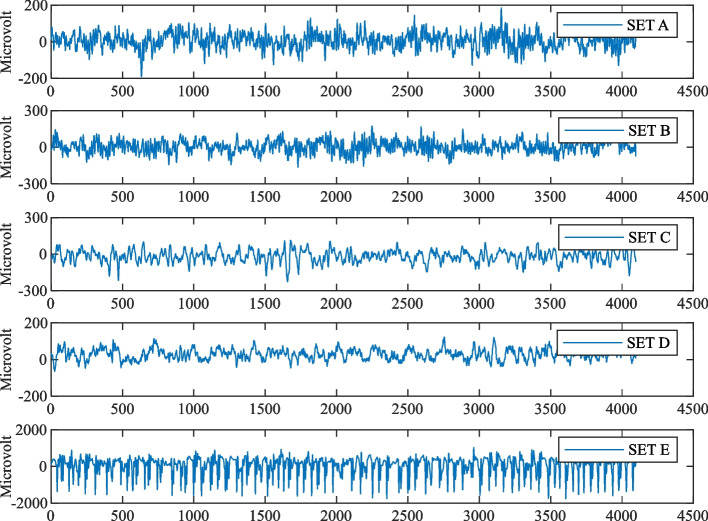
Example of a 5-class EEG signal

Figure 1 shows the visual graphics of the data fragments of Group 1 in each subset, where the horizontal axis represents the number of samples of EEG time series and the vertical axis represents the sample value. It can be seen that there are some differences in the 5 types of EEG signal waveforms. Due to the presence of feature waves for epileptic EEG signals, the EEG signal amplitude of Set E is significantly larger than those of the other four groups.

#### New Delhi EEG dataset

These datasets were exemplary segmented EEG time series recordings of 10 epilepsy patients from the Neurology & Sleep Centre, Hauz Khas, New Delhi. The datasets were acquired using the Grass Telefacor Comet AS40 amplification system at a sampling frequency of 200 Hz. Gold-plated scalp EEG electrodes were placed using a 10–20 electrode placement system at the time of acquisition. The acquired EEG signal is filtered by a band-pass filter from 0.5 Hz ~ 70 Hz. There are three states including preictal, interictal and ictal, which are in the form of MAT. Each EEG state contains 50 MAT files, and each MAT file consists of 1024 samples of one EEG time series data with a duration of 5.12 s.

### Research methods

**Fig. 2:**
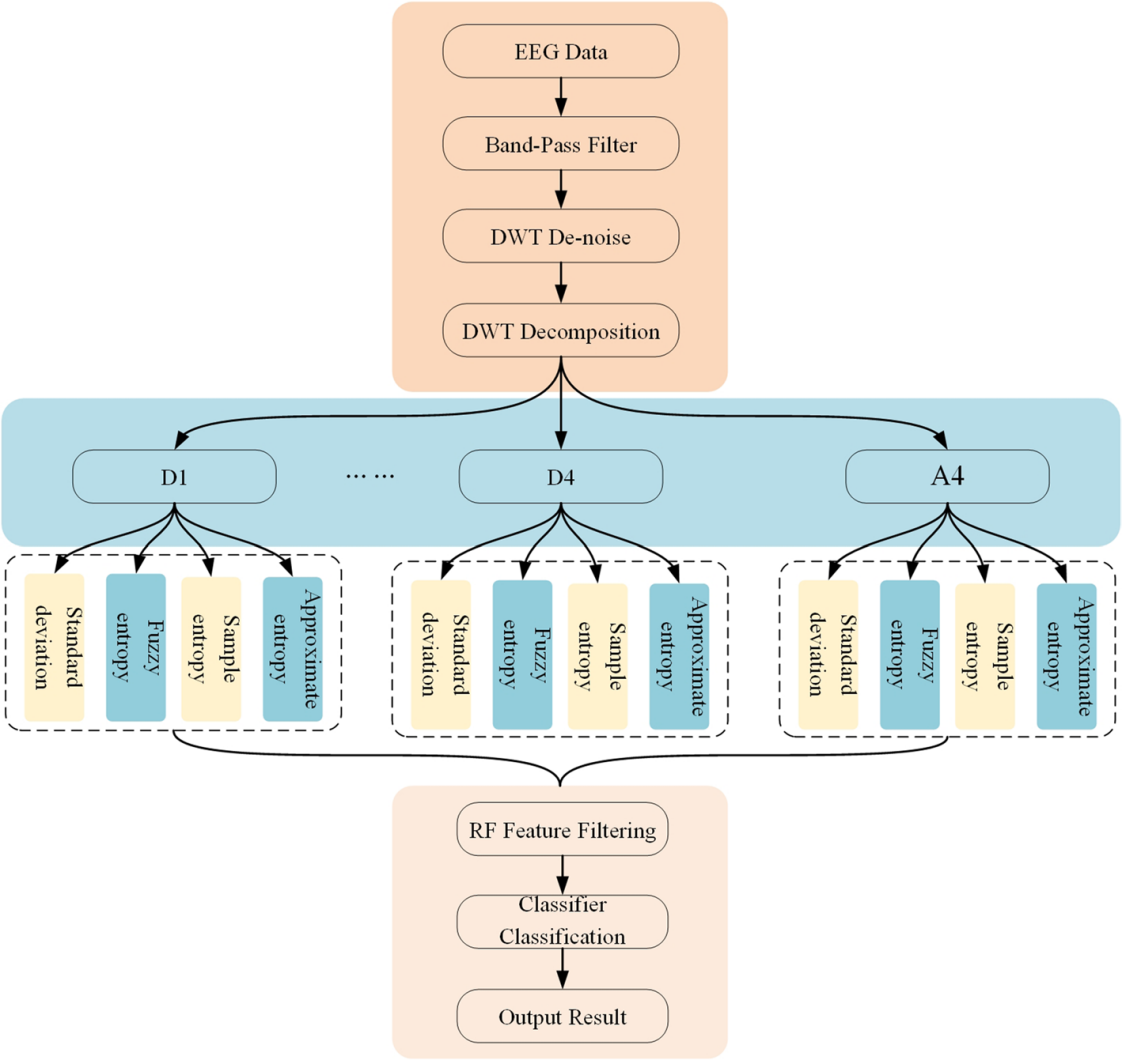
Epilepsy classification flowchart

The study process in this paper is divided into four steps. Firstly, it is necessary to preprocess the EEG signal data, where it is filtered through the bandpass filter, and then DWT is used to decompose and reconstruct the wavelet to realize wavelet denoising. Secondly, feature extraction is performed. The reconstructed EEG signal is decomposed again, which is divided into five subbands D1, D2, D3, D4, and A4, and four types of features including time domain standard deviation (STD), ApEn, FuzzyEn, and SampEn are extracted from the above five subbands. Thirdly, feature selection is carried out. The random forest algorithm is adopted to evaluate the importance of features and select the most important 10 features. Finally, the fourth step is classification, using CNN to classify EEG signals. The method block diagram is shown in Fig. 2.

### Data preprocessing

In order to improve the accuracy of subsequent feature extraction and classification, it is necessary to filter and denoise the EEG signals. The feature wave of epilepsy EEG signal covers the 0 ~ 80 Hz frequency band, while the sampling frequency of the experimental dataset is 173.61 Hz, so the 4th-order Butterworth bandpass filter is used to obtain an EEG signal of 0.01 Hz ~ 86.8 Hz. The filtered EEG was decomposed by using the "db4" wavelet basis function, and the select threshold was selected for denoising. Then the denoised subband was reconstructed to obtain the filtered denoised EEG.

The Fourier transform, which is traditionally used for the Joint Time–Frequency Analysis of signals, only can process stationary signals, while wavelet transforms can process non-stationary complex signals such as EEG signals. Therefore, the EEG signal preprocessing and EEG signal decomposition were realized using DWT in this paper. The DWT was used to denoise the raw EEG data. The EEG signal was decomposed by multi-level wavelet decomposition, and the approximation coefficient and detail coefficient of the signal at various scales were obtained.

It is assumed that the function *φ*(*t*) is a quadratic integral function which is denoted as *φ*(*t*) ∈ *L*^2^(**R**), where *L*^2^(**R**) represents the square-integrable space of real numbers. Its Fourier Transform *Ψ*(*ω*) satisfies the following equation:

1. $${C}_{\Psi }={\int }_{-\infty }^{+\infty }\frac{{\left|\Psi \left(\omega \right)\right|}^{2}}{|\omega |}\mathrm{d}\omega <\infty$$

The continuous wavelet function *Ψ*_*s,t*_ (*t*) is obtained from the fundamental wavelet *Ψ*(*t*) by scale scaling and translation, which is expressed as:

2. $${C}_{\Psi }={\int }_{-\infty }^{+\infty }\frac{{\left|\Psi \left(\omega \right)\right|}^{2}}{|\omega |}\mathrm{d}\omega <\infty$$

where *s* is the scale factor, *τ* is the translation factor, and **R** represents the set of real numbers.

Next, the discretizations of the scale factor and translation factor are performed. Assuming that *s* = 2^−*j*^ and *τ* = *k*2^−*j*^, where *j* and* k* are the size of the scaling and the translation scale, respectively, and the values of *j* and *k* are integers. And then, the expression of the discrete wavelet function for the *Ψ*(*t*) can be written as:

3. $${\Psi }_{{2}^{-j},k{2}^{-j}}\left(t\right)={2}^{j/2}\Psi \left({2}^{j}t-k\right)$$

For any function *f*(*x*), the DWT can be expressed as:

4. $${W}_{\Psi }f(j,k)={2}^{j/2}{\int }_{-\infty }^{+\infty }f\left(t\right){\Psi }^{*}\left({2}^{j}t-k\right)\mathrm{d}t$$

**Fig. 3:**
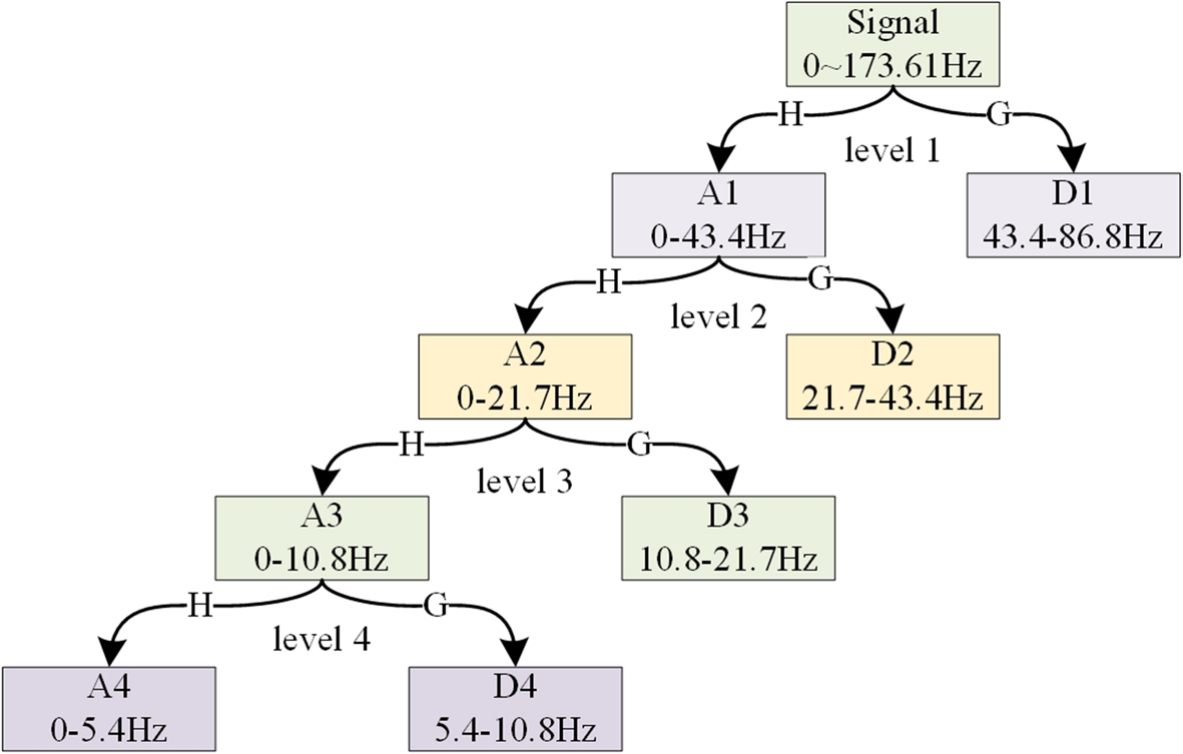
4-level DWT decomposition of EEG signal

In this study, the input signal passes through the low-pass filter *G*(*n*) and the high-pass filter *H*(*n*), both of which have a cut-off frequency of one-quarter of the sampling frequency. In the first step of DWT decomposition, the low-frequency approximation coefficient A1 and detail coefficient D1 are obtained, and then, the output A1 is fed to another quadrature mirror filter. By means of repeating the same process, the approximation and detail coefficient for the next level can be obtained. Considering that the frequency band above 80 Hz may not contain the eigenwaves of epileptic EEG, the "db4" wavelet basis function was used to perform a 4-level decomposition of EEG signals. Figure 3 illustrates the 4-level decomposition of EEG signals. The subband frequencies of A1, D1, A2, D2, A3, D3, A4, and D4 are 0 ~ *f*_*s*_/4, *f*_*s*_/4 ~ *f*_*s*_/2, 0 ~ *f*_*s*_/8, *f*_*s*_/8 ~ *f*_*s*_/4, 0 ~ *f*_*s*_/16, *f*_*s*_/16 ~ *f*_*s*_/8, 0 ~ *f*_*s*_/32, *f*_*s*_ /32 ~ *f*_*s*_/16, 0 ~ *f*_*s*_*/*64, *f*_*s*_/64 ~ *f*_*s*_ /32, respectively, where fs is the sampling frequency of the used data set, being 173.61 Hz.

### Feature extraction

Firstly, DWT wavelet decomposition is performed on the filtered denoised EEG signals. The "db4" was selected as the wavelet basis function, and the 4-stage decomposition was used to obtain five subbands of D1 ~ D4 and A4. Then, the multiple features of the STD, SampEn, FuzzyEn, and ApEn were extracted from the EEG signals of the above five subbands. The extracted 20 EEG features are shown in Table 1.

**Table 1:** EEG features of epilepsy

D1STD	D2 STD	D3 STD	D4 STD	A4 STD
D1 SampEn	D2 SampEn	D3 SampEn	D4 SampEn	A4 SampEn
D1 ApEn	D2 ApEn	D3 ApEn	D4 ApEn	A4 ApEn
D1 FuzzyEn	D2 FuzzyEn	D3 FuzzyEn	D4 FuzzyEn	A4 FuzzyEn

### Nonlinear features

With an in-depth understanding of EEG signals, it is generally believed that human EEG signals are nonlinear random signals in the field of bioelectric signals, and their nonlinear features can better characterize EEG signals. Entropy is a physical quantity that can characterize the EEG complexity. Studies have shown that the uncertainty of EEG signals during the ictal phase is significantly reduced, so it is necessary to characterize the features of EEG signals using entropy. ApEn was developed on the basis of Kolmogorov-Sinai entropy and was proposed by Pincus in 1991 [34]. ApEn predicts the amplitude of the future signal based on the known signal amplitude, which can be used to describe the uncertainty or randomness of the signal. SampEn was proposed by Richman et al. [35]. The SampEn is similar to the ApEn in the physical meaning, but the SampEn overcomes three following shortcomings of the ApEn: SampEn removes the self-match from the data. SampEn obtains the total number of well-matched templates before the logarithmic operation. When dimension *m* is embedded, the reconstructed time series in SampEn is *N*-*m* rows instead of *N*-*m* + 1 rows of ApEn, so that the number of patterns in embedding dimension m and *m* + 1 are equal. FuzzyEn characterizes the occurrence probability of the new pattern, and the larger the measured value, the greater the occurrence probability of the new pattern, that is, the greater the complexity of the sequence.

### Standard Deviation (STD)

Since the STD can achieve a good recognition effect, as a simple and computable time–frequency feature, the STD is also applied to EEG signals in this paper. The calculation formula of the STD *σ* is defined as:

5. $$=\sqrt{\frac{{\sum_{i=1}^{N}\left({x}_{i}-\overline{x }\right)}^{2}}{N}}$$

where *x* represents the average of *x*_*i*_. *N* is the total sample quantity, and* x* is a variable.

### Feature selection

In this paper, the random forest algorithm was used to evaluate the extracted 20 EEG signal features importance and sorted them in descending order. According to the feature importance, the last feature in each round was removed. Thus, a new feature set is obtained and the above process is repeated with the new feature set, and the process does not stop until the 10 features with the highest importance are left.

**Fig. 4:**
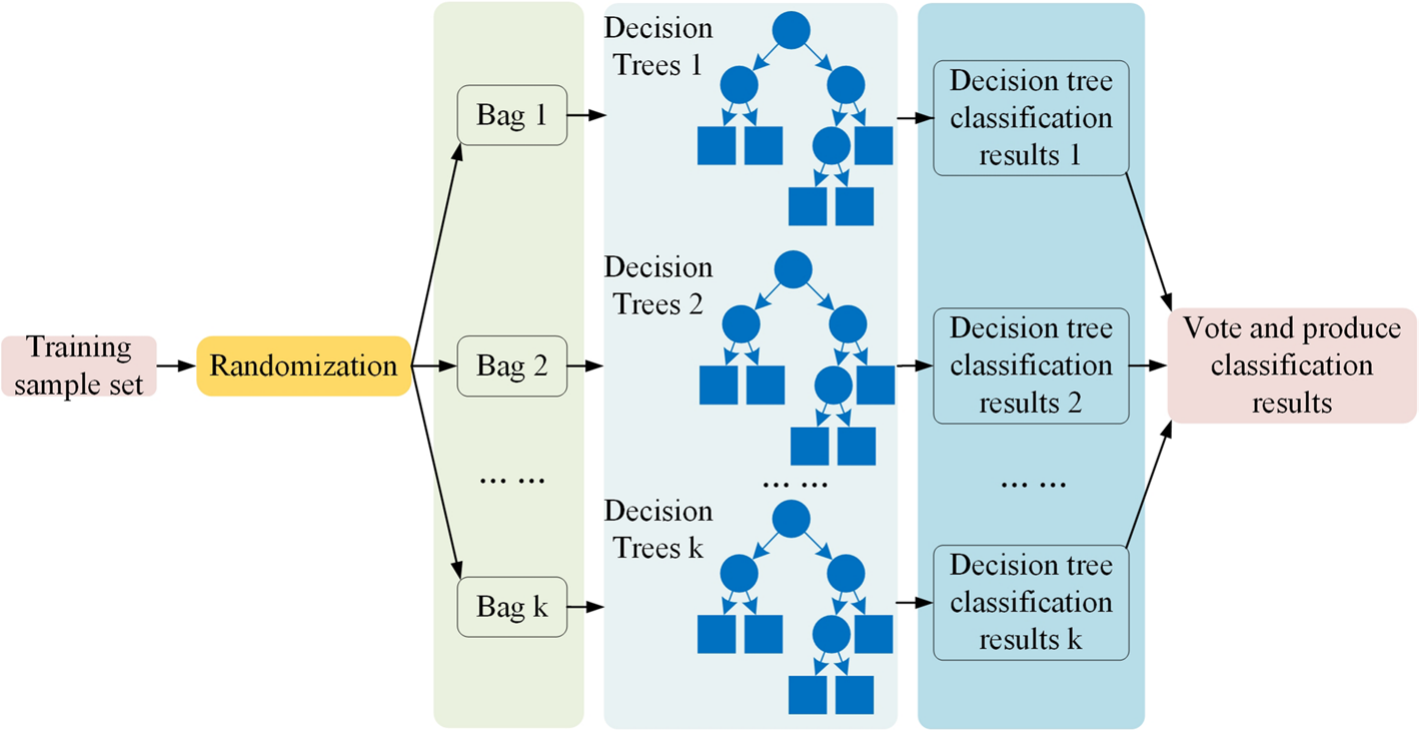
RF model

As an ensemble learning algorithm, Random Forests (RF) uses decision trees as the basic unit. The decision trees are added into RF on the basis of Bagging, which is an improved version of the Bagging algorithm. The training of RF subsets is independent of each other and efficient. It also retains the advantages of the Classification and Regression Tree (CART) algorithm, which uses Gini coefficients to select the optimal features and syncopation point, and overcomes the disadvantages of CART which require a fully spanning tree. The operation principle of random forest is shown in Fig. 4.

For RF, *k* samples are taken from the dataset using bootstrap sampling, and each sample has *N* features. Then *k* decision models are established for each of the *k* samples, and the *k-*th decision tree is labeled as *T*_*k*_. The *k-*th bootstrap sample was trained to calculate the classification accuracy of the *k-*th Out of bag (OOB) data *LOOB k*. The feature *X*_*j*_ (*j* = 1,2,…, *N*) in the OOB data was disturbed randomly, and the classification accuracy was calculated again. And then, the above process is repeated when *k* = 2, 3, 4, …, in order. The importance of the feature *P*_*j*_ is calculated by the following equation.

6. $${P}_{j}=\frac{1}{K}\sum_{j=1}^{K}\left({L}_{k}^{OOB}-{L}_{k,j}^{OOB}\right)$$

Finally, they are ranked according to their importance and the features with the lowest importance are excluded.

### Classification

**Fig. 5:**
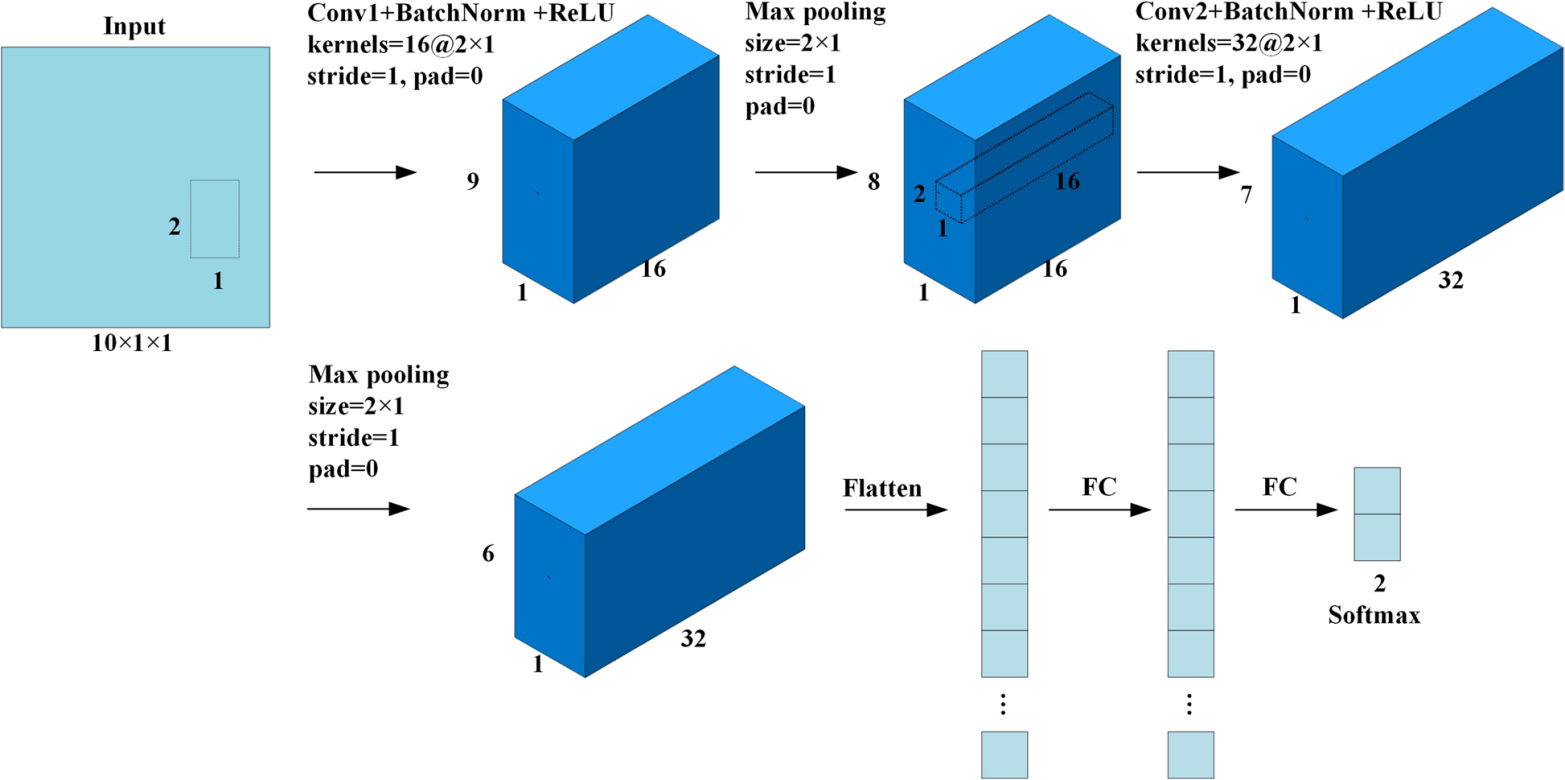
The architecture of the convolutional neural network used in this work

In this work, the CNN architecture is defined with 16 filters of size 2 × 1 with a stride of 1 for the first convolutional layer. An input data of 10 × 1 × 1 was used as input to this convolutional layer. After the first convolutional layer, batch normalization and max-pooling were performed using a filter of 2 × 1 with a stride of 1. Again, for the next convolutional layer, 32 filters of size 2 × 1 were used with a stride of 1. Similarly, batch normalization and max-pooling were performed using a filter of size 2 × 1 and a stride of 1 after the second convolutional layer. There are two fully connected layers that use softmax as the activation function after the two convolutional layers. Adaptive Moment Estimation (Adam) is used to learn the parameters of the CNN. The dataset used in the experiment was divided into a training and test set with a ratio of 3:1, and the CNN classifier was used to classify the selected feature data. The CNN architecture diagram is shown in Fig. 5.

The convolutional layer consists of several convolutional units, and the parameters of each convolutional unit are optimized by a backpropagation algorithm. The different features of the input are extracted by convolution, which is calculated as follows.

7. $${H}_{i,j}=f{\left(C{D}^{k}*x\right)}_{i,j}+{a}_{k}$$

where* f* is the activation function, *D*^*k*^ is the *K-*th convolution kernel, *a*_*k*_ is the offset error for the sum of the results of the *K-*th convolution kernel, and *x* is the convolution input data.

The pooling layer, also called the downsampling layer, mainly subsamples the feature maps learned in the convolutional layer, which reduces the input dimension of the subsequent network layers, and improves the computational accuracy.

The average pooling can be expressed as:

8. $$y\left(x\right)=\frac{1}{k*k}\sum_{i={i}_{1}}^{{i}_{1+k}}\sum_{j={j}_{1}}^{{j}_{1+K}}{x}_{i,j}$$

The max pooling is given as:

9. $$\left(x\right)=max\left({X}_{[i,i+k][j,j+k]}\right)$$

The fully connected layer is fully connected by using softmax, and the obtained activation values are the features extracted by the convolutional neural network, and the features learned by the convolutional layer and the pooling layer are weighted and fused to the sample labeling space.

## Results and discussion

### Evaluation metrics

To evaluate the performance of the model, Accuracy, Sensitivity, Specificity, and Precision metrics are used in this paper. The indicators are calculated as follows:

10. $$Accuracy=\frac{TP+TN}{TP+FN+FP+TN}$$

11. $$Sensitivity=\frac{TP}{TP+FN}$$

12. $$Specificity=\frac{TN}{TN+FP}$$

13. $$Precision=\frac{TP}{TP+FP}$$

where *TN* is the true negative rate, which indicates the number of samples that are actually negative samples predicted to be negative samples; *FP* is the false positive rate, which indicates the number of samples that are actually negative samples predicted to be positive samples; *FN* is the false negative rate, which indicates the number of samples that are actually positive samples predicted to be negative samples; *TP* is the true positive rate, which represents the number of samples that are actually positive samples predicted to be positive samples.

### Experimental results

**Fig. 6:**
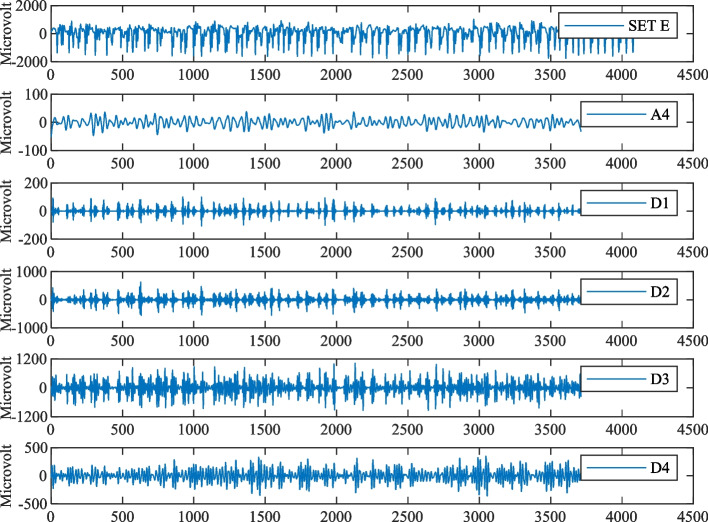
The subband waveforms of Set E decomposed by DWT

**Fig. 7:**
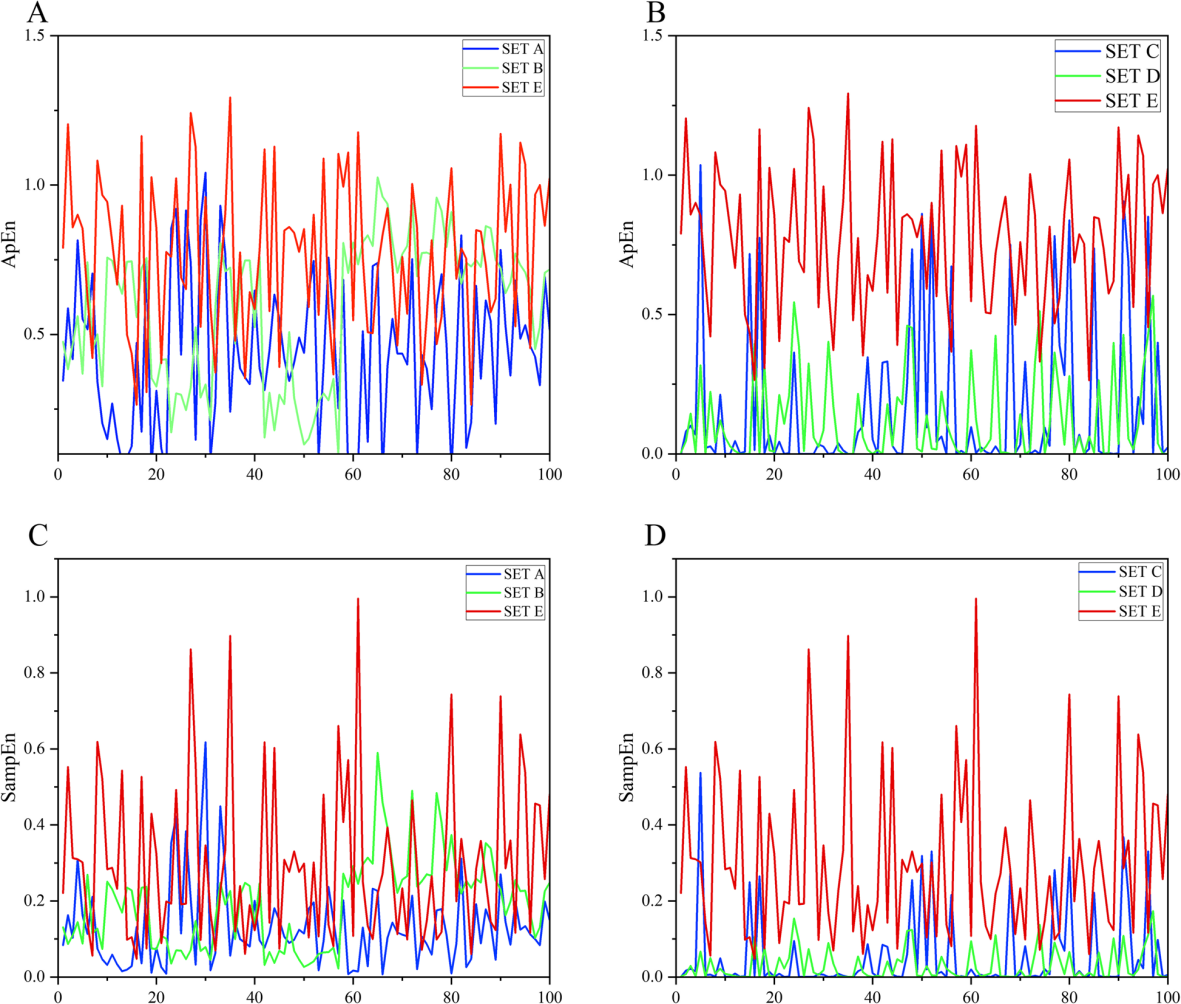
**A**, **B** the ApEn feature of D1 subband, **C**, **D** the SampEn feature of D1 subband

**Fig. 8:**
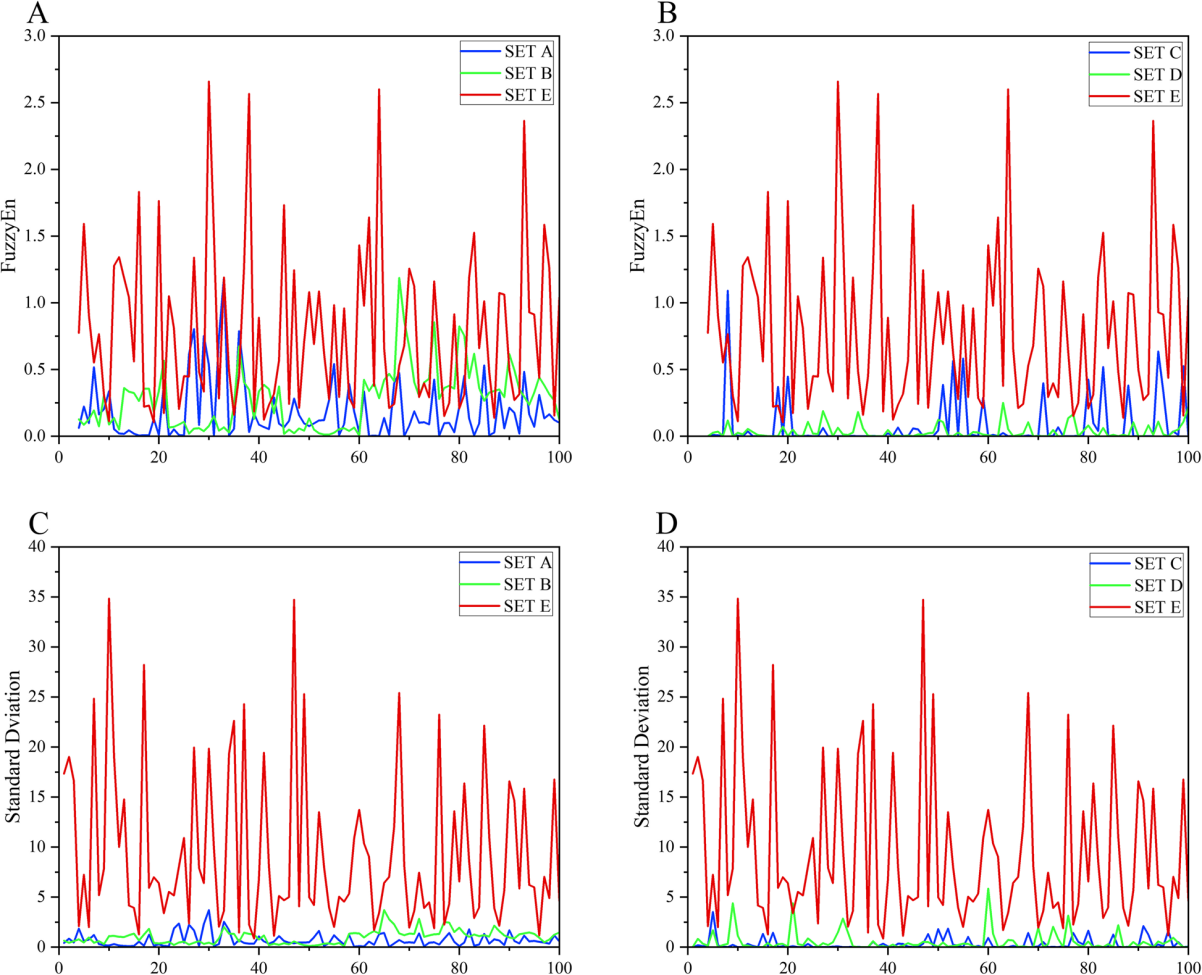
**A**, **B** the FuzzyEn feature of D1 subband, **C**, **D** the STD feature of D1 subband

In order to extract the features of the EEG signals effectively, the wavelet decompositions for Set A, Set B, Set C, Set D, and Set E of the Bonn EEG dataset were carried out. Taking the Set E subset during the ictal period as an example, the DWT was adopted to perform a 4-level wavelet decomposition. The subband waveforms of Set E decomposed by DWT are shown in Fig. 6, where the horizontal axis represents the number of samples of EEG time series and the vertical axis represents the sample value. The subband frequencies of A4, D4, D3, D2 and D1 are 0 ~ 5.4 Hz, 5.4 ~ 10.8 Hz, 10.8 ~ 21.7 Hz, 21.7 ~ 43.4 Hz, and 43.4 ~ 86.8 Hz, respectively. And then, the effective features for all the subbands were extracted and analyzed. For convenience, the analysis of features including ApEn, FuzzyEn**,** SampEn, and STD features for decomposed D1 subband was given in this paper. The extracted features for the D1 subband are shown in Figs. 7 and 8, where the horizontal axis represents each data segment and the vertical axis represents the feature value of each data segment. For the D1 subband, there are significant differences in the amplitudes of the above four features. The amplitude of the four features of the D1 subband in the inter epileptic Set D is significantly lower than those in Set A and E, so the classification effect could be greatly improved by using the four features to classify the interictal period and the ictal period or healthy people. For Set A and Set E, the FuzzyEn feature amplitude and STD feature amplitude are quite different, so the two features can play very important roles in the classification of Set A and Set E. For the approximates entropy and SampEn features, most of the feature amplitudes for Set E are lower than those for Set A, while there is a small overlap. Therefore, it is necessary to use random forest-based feature selection to remove the poor features, and the adopted 10 features with the best importance are shown in Table 2.

**Table 2:** The adopted 10 most important features

D1 SampEn	D1 ApEn	D4 ApEn	A4 FuzzyEn	D1 FuzzyEn
D2 FuzzyEn	D4 FuzzyEn	A4 STD	D4 STD	D3 STD

**Fig. 9:**
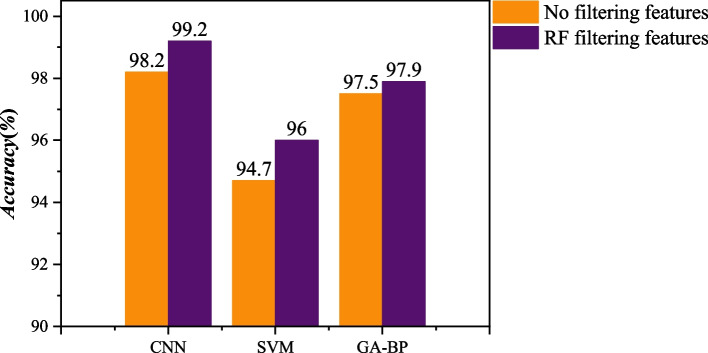
Prediction results with feature filtering

In order to more intuitively reflect that it is essential to perform feature selection, the compared experiment before and after performing feature selection was carried out. The data was divided into a training set and a test set with a ratio of 3:1, and MATLAB R2019A was employed to construct and simulate the model. For the classification results between Set D and Set E EEG signals, the accuracies obtained by CNN, SVM, and BP neural network optimized by genetic algorithm (GA-BP) classifiers without feature screening are 98.2%, 94.7%, and 97.5%, respectively. When the 10 features obtained from the screening are used for classification, the accuracies of CNN, SVM,  and GA-BP classifiers are improved to be 99.2%, 96%, and 97.9%, respectively. Figure 9 shows the compared results for the classification between Set D and Set E EEG signals. It can be seen that for CNN, SVM, and GA-BP, using a random forest algorithm to screen the importance of features can improve classification accuracy. Especially, the classification accuracy rate of the CNN algorithm can be improved to 99.2% after feature selection.

**Fig. 10:**
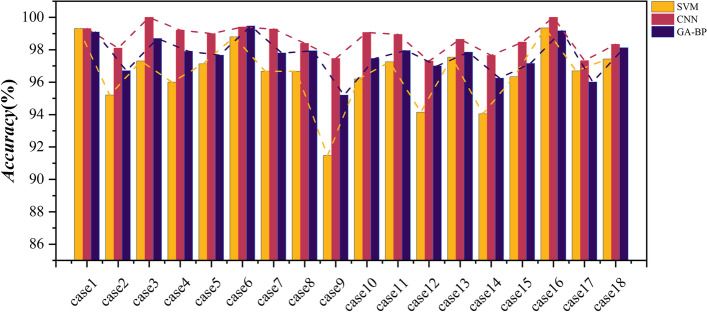
Accuracy results of SVM, CNN, and GA-BP classifiers

**Fig. 11:**
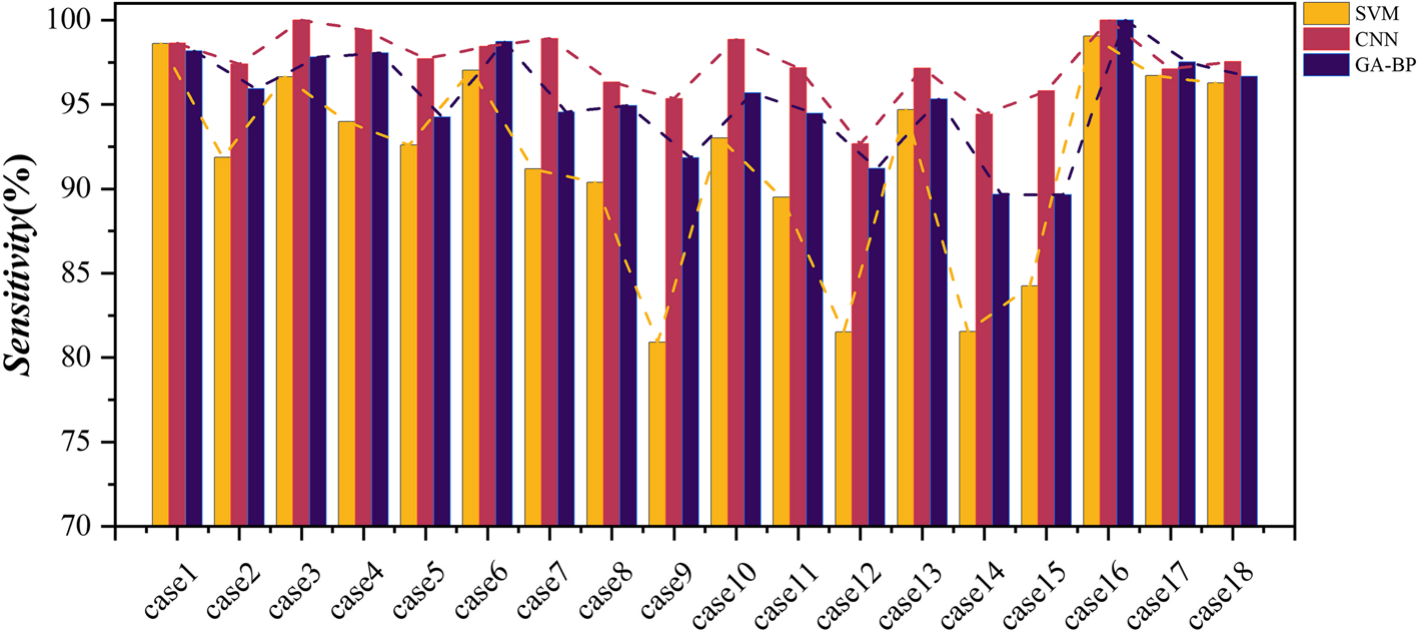
Sensitivity results of SVM, CNN, and GA-BP classifiers

**Fig. 12:**
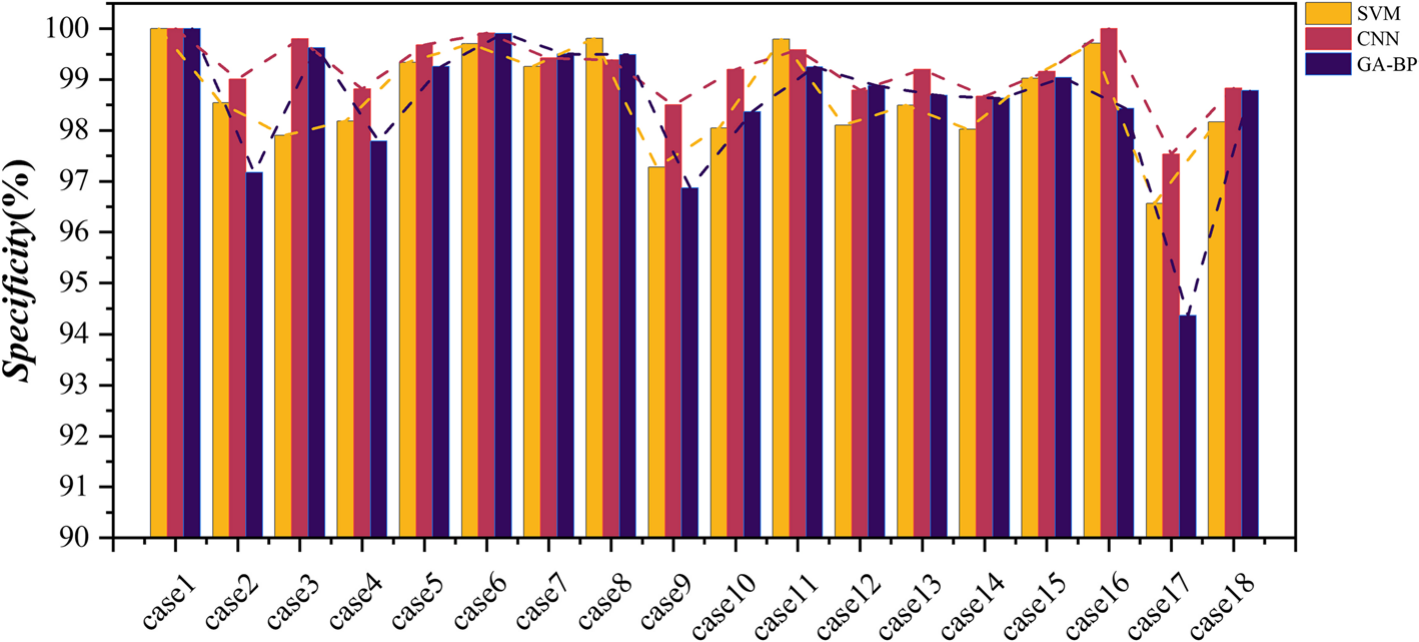
Specificity results of SVM, CNN, and GA-BP classifier

**Fig. 13:**
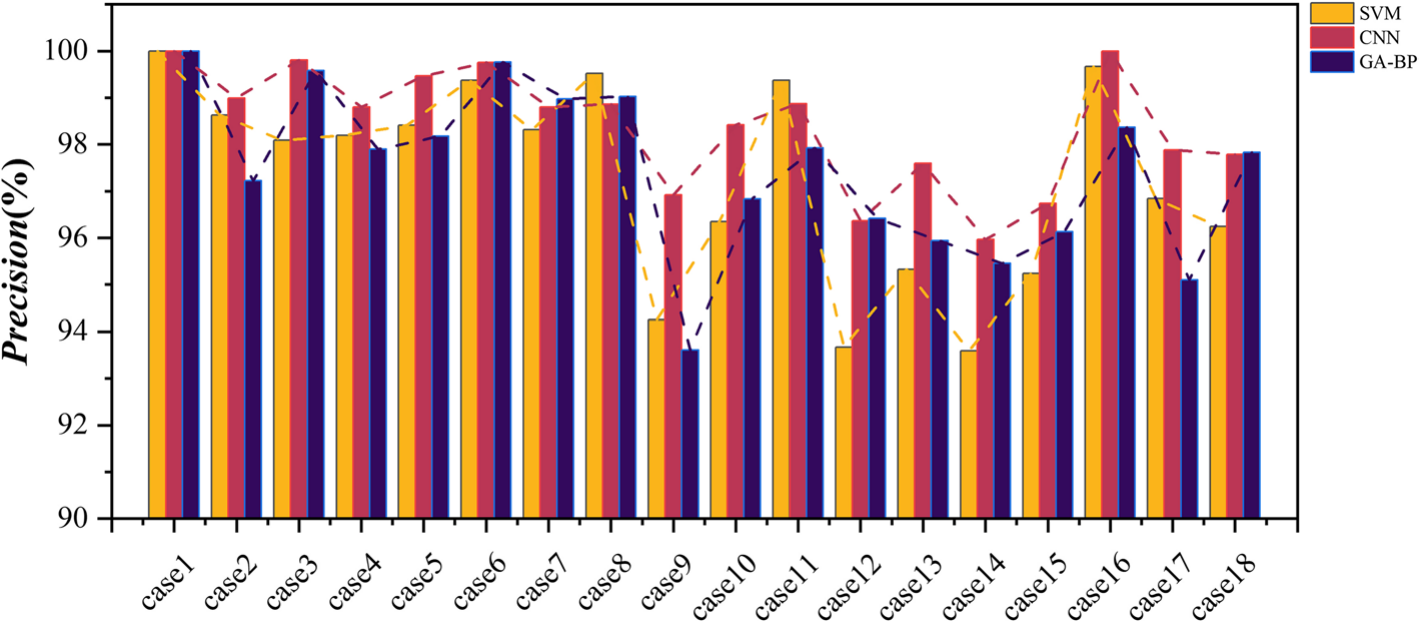
Precision results of SVM, CNN, and GA-BP classifiers

In order to verify the superiority of the CNN algorithm in classification applications, SVM and GA-BP classifiers along with CNN classifiers were also employed. Table 3 describes the classification tasks in this topic. And the Accuracy, Sensitivity, Specificity, and Precision of the binary classification task are listed in Table 4. Moreover, to visually show the superiority of the CNN classifier, Figs. 10, 11, 12, and 13 shows the Accuracy, Sensitivity, Specificity, and Precision, respectively, where the horizontal axis represents the different cases and the vertical axis represents the value of index. The experimental results show that the classification accuracy obtained by the classification algorithm combining RF and CNN is much higher than that of combining RF with SVM and GA-BP.

**Table 3:** The specific classification tasks

Classification Task	Case Number	Dataset	Lable
Healthy Vs. Epileptic	Case1	Bonn (A-E)	Datasets A, B, C, and D are labeled as 0, dataset E is labeled as 1
	Case2	Bonn (B-E)	
Interictal Vs. Ictal	Case3	Bonn (C-E)	
	Case4	Bonn (D-E)	
Healthy Vs. Epileptic	Case5	Bonn (AB-E)	
Nonictal Vs. Ictal	Case6	Bonn (AC-E)	
	Case7	Bonn (AD-E)	
	Case8	Bonn (BC-E)	
	Case9	Bonn (BD-E)	
Interictal Vs. Ictal	Case10	Bonn (CD-E)	
Nonictal Vs. Ictal	Case11	Bonn (ABC-E)	
	Case12	Bonn (ABD-E)	
	Case13	Bonn (ACD-E)	
	Case14	Bonn (BCD-E)	
	Case15	Bonn (ABCD-E)	
Interictal Vs. Ictal	Case16	New Delhi (Interictal-Ictal)	The dataset of Preictal and Interictal are labeled as 0, and the dataset of Ictal is labeled as 1
Preictal Vs. Ictal	Case17	New Delhi (Preictal-Ictal)	
Nonictal Vs. Ictal	Case18	New Delhi (Nonictal-Ictal)

**Table 4:** Accuracy, sensitivity, specificity, and precision results of SVM, CNN, and GA-BP classifiers

	**Accuracy (%)**			**Sensitivity (%)**			**Specificity (%)**			**Precision (%)**		
	CNN	SVM	GA-BP	CNN	SVM	GA-BP	CNN	SVM	GA-BP	CNN	SVM	GA-BP
case1	99.30	99.30	99.10	98.62	98.62	98.18	100	100	100	100	100	100
case2	98.10	95.20	96.70	97.39	91.86	95.92	99.01	98.54	97.18	98.99	98.63	97.22
case3	99.90	97.30	98.70	100	96.64	97.81	99.80	97.90	99.62	99.81	98.09	99.58
case4	99.20	96.00	97.90	99.42	93.98	98.04	98.82	98.18	97.79	98.80	98.20	97.90
case5	99.00	97.13	97.67	97.71	92.59	94.24	99.68	99.34	99.25	99.47	98.41	98.18
case6	99.40	98.80	99.47	98.45	97.02	98.72	99.91	99.70	99.91	99.75	99.38	99.76
case7	99.28	96.67	97.80	98.91	91.17	94.55	99.42	99.26	99.50	98.80	98.31	98.98
case8	98.40	96.67	97.93	96.32	90.38	94.94	99.38	99.81	99.49	98.86	99.52	99.03
case9	97.46	91.47	95.20	95.35	80.91	91.85	98.50	97.28	96.87	96.92	94.25	93.61
case10	99.07	96.20	97.47	98.85	93.00	95.69	99.20	98.05	98.37	98.42	96.34	96.83
case11	98.95	97.25	97.95	97.17	89.50	94.47	99.59	99.80	99.25	98.87	99.37	97.92
case12	97.30	94.15	97.00	92.66	81.51	91.21	98.79	98.11	98.88	96.37	93.67	96.42
case13	98.65	97.53	97.85	97.14	94.69	95.32	99.20	98.49	98.69	97.59	95.32	95.94
case14	97.65	94.05	96.25	94.43	81.56	89.67	98.67	98.02	98.63	95.96	93.59	95.45
case15	98.47	96.33	97.16	95.80	84.24	89.64	99.16	99.03	99.04	96.74	95.24	96.13
case16	100	99.34	99.17	100	99.04	100	100	99.71	98.43	100	99.67	98.37
case17	97.33	96.69	96.01	97.10	96.72	97.51	97.53	96.56	94.36	97.88	96.84	95.10
case18	98.33	97.43	98.12	97.53	96.27	96.65	98.83	98.17	98.78	97.79	96.24	97.83

## Discussion

In order to verify the advantages of the proposed model over other classification techniques, we compare the results obtained by other methods with our proposed methods, which are shown in Table 5. So as to make the calculation results more comparable, only the results obtained from using the same data set and similar cases are listed in this paper. The classification model proposed in this paper can achieve an accuracy of 99.9%, a sensitivity of 100%, a precision of 99.81%, and a specificity of 99.8% in the binary classification task of interictal and ictal periods of Bonn EEG datasets. In terms of New Delhi EEG datasets can achieve an accuracy of 100%, a sensitivity of 100%, a precision of 100%, and a specificity of 100% in the binary classification task of interictal and ictal periods. It can be seen that the RF + CNN algorithm used in this paper for mixed features is considered to be a noteworthy improvement compared to state-of-the-art methods. For the classification of Set D and Set E, Wang et al. [21] utilized a Short Time Fourier Transform (STFT), average energy, and Principal Component Analysis (PCA) feature as the basis for classification, and random forest-grid search optimization (RF + GSO) for the extracted features. Jaiswal and Banka [24], Riaz et al. [6], and Deepti Tripathi and Agrawal [13] proposed an automatic classification technique based on SVM. Xin et al. [36] proposed an Attention Mechanism-based Wavelet Convolution Neural Network (AMWCNN) for epilepsy EEG classification. However, our proposed RF + CNN model system is superior to their method. Jiang et al. [37] used Wavelet Packet Decomposition (WPD) to extract features from EEG and adopted Takagi Sugeuo Kang (TSK) classifier to classify epileptic status. Lu et al. [22]. proposed Kraskov entropy and instantaneous area as features to classify interictal signals and ictal signals using the LS-SVM classifier. Al-Hadeethi et al. [38] recommended the method that the multiple time-domain features combined with Kolmogorov Smirnov Test (KST) are used for feature selection and AdaBoost is used for classification. Although these studies on the classification of interictal and ictal signals have yielded encouraging results, their classification accuracy is lower than the model that we proposed.

**Table 5:** Comparison of seizure detection methods using the benchmark Bonn EEG dataset

Article	Year	Selected features	Classifier	Case	Accuracy (%)
Riaz et al. [6]	2016	Time matrix + spectral features	SVM	A-E D-E	97.00 92.00
Raghu et al. [14]	2017	Wavelet Packet norm Entropy	REN	C-E	99.60
Jiang et al. [37]	2017	WPD	TSK	A-E	91.40
Jaiswal and Banka [39]	2017	EEG	1D-Local Gradient Patterns (LGP) + SVM	C-E D-E	99.10 99.07
Jaiswal et al. [24]	2018	PCA	SVM	D-E ABCD-E	95.50 97.40
Tripathi and Agrawal [13]	2018	FuzzyEn	SVM	C-E D-E	98.62 97.00
Lu et al. [22]	2018	Kraskov entropy + instantaneous area	LS-SVM	C-E D-E	99.00 97.00
Wang et al. [21]	2019	STFT + average energy + PCA	RF + GSO	C-E D-E	98.50 98.10
Zhao and Wang [31]	2020	EEG	CNN	D-E	98.50
Shoeibi et al. [40]	2021	Timedomain + Power spectrum + Nonlinear features + Lyapunov index	Fisher + CNN	C-E	96.67
Banupriya and Devi [20]	2021	EEG	VSPO-SVM	D-E	98.13
Al-Hadeethi et al. [38]	2021	Max + Min + Mode + range + var + standard deviation	KST + AdaBoost	C-E AB-E CD-E	98.50 98.00 98.20
Aayesha et al. [29]	2022	Time domain + spectrum + nonlinear features + Local Binary Pattern	Feedforward Neural Network	A-E B-E C-E D-E AB-E CD-E ABCD-E	96.67 91.67 91.67 85.00 90.00 91.11 90.67
Xin et al. [36]	2022	DWT decompose EEG	AMWCNN	C-E D-E	99.39 99.11
Hemachandira and Viswanathan [7]	2022	DWT Haar + db4 + Sym8	Particle Swarm Optimization (PSO) + SVM	A-E	98.00
Proposed study	2022	Time–frequency + nonlinear features	RF + CNN	A-E B-E C-E D-E AB-E AC-E AD-E BC-E BD-E CD-E ABC-E ABD-E ACD-E BCD-E ABCD-E	99.30 98.10 99.90 99.20 99.00 99.40 99.28 98.40 97.46 99.07 98.95 97.30 98.65 97.65 98.47

## Conclusion

Accurate classification may reduce the damage caused by seizures. In this paper, we propose a novel epileptic EEG signal classification methodology using a multivariate feature classification method based on the combination of RF and CNN to classify different epileptic states (i.e., nonictal, preictal, interictal, and ictal). The method is verified by the multichannel EEG signals in the Bonn database and New Delhi database. It can be concluded through the study that: (1) the proposed EEG signal classification method outperforms other benchmark models in classifying different epileptic states; For the C-E case, the proposed model achieves a classification accuracy of 99.9%, a sensitivity of 100%, a specificity of 99.80%, and a precision of 99.81%. For the interictal-ictal case of New Delhi datasets, the proposed model achieves a classification accuracy of 100%, a sensitivity of 100%, a specificity of 100%, and a precision of 100%. (2) the proposed method can extract multiple features from EEG signals; (3) The RF + CNN model can be used to rank the extracted EEG features according to their importance and achieve feature selection, so as to achieve higher classification accuracy. In medicine, the proposed EEG classification method has important practical significance for the diagnosis and treatment of epilepsy. For example, for patients, the high classification accuracy of epileptic states classified by EEG signals (i.e., interictal, ictal) can achieve reliable and timely early warning; For doctors, it can help them understand the classification of epilepsy in patients so that the prevention and treatment of epilepsy can be effectively controlled.

Thus, this work addresses one important challenges of accurately classifying epileptic states by multi-feature EEG signals. As part of our future research, we aim to improve EEG classification methods in the following ways to better serve the prevention and treatment of epilepsy: (1) the proposed EEG classification model will be used to detect seizures; (2) through combining with the temporal correlation between EEG signal frames, the false detection of seizures may be further reduced, however, further studies need to be performed.

## Data Availability

Publicly available dataset was analyzed in this study and the dataset can be freely accessed. The datasets we used in our work can be found as the following link https://github.com/RYH2077/EEG-Epilepsy-Datasets, and further inquiries can be directed to the corresponding author.
